# Energy-based method for designing aptamers to target phosphatidylserine

**DOI:** 10.1016/j.mex.2026.103872

**Published:** 2026-03-19

**Authors:** Fatima Alharbi, Hamed Alsulami, Suliman AlOmar, Md. Ashrafuzzaman

**Affiliations:** a Biochemistry Department, College of Science, King Saud University, Riyadh, Saudi Arabia; b Doping Research Chair, Zoology Department, College of Science, King Saud University, Riyadh, Saudi Arabia; c Doping Research Chair, Deanship of Scientific Research, King Saud University, Riyadh, Saudi Arabia

**Keywords:** Aptamer, Phosphatidylserine, Screened Coulomb interaction, Energetics, Diagnostics, Therapeutics

## Abstract

Among existing aptamer design methods, none can address aptamer-target binding energetics during the design phases. A novel aptamer design platform has been proposed to overcome this issue by combining theoretical and computational methods. This is primarily an energy-based platform, focusing on calculating drug-target binding energies (DTBEs) and monitoring the phenomenological energetics of drug-target binding. This relates to understanding the statistical significance of drug-target association/dissociation processes, considering a screened Coulomb interaction (SCI) formalism applied among a distribution of functional charges in a drug-target complex. The interactions of a whole aptamer or any aptamer building block (ABB) (adenine, guanine, cytosine, thymine, or uracil) with the optimal target molecule are considered to calculate DTBEs. Due to various important diagnostic and therapeutic roles in diseases, we chose phosphatidylserine (PS) as our biological target and designed a set of aptamers for it. Using the same technique, concomitantly, we succeeded in discovering aptamers for both lipid and protein biomolecules of therapeutic interest. An aptamer is constructed using a seed-and-grow approach, optimizing SCIs, and selecting the aptamer length based on the trend of DTBE reaching towards equilibrium in a biological environment. This novel aptamer design platform, the ‘screened Coulomb interaction approach (SCIA)’, ensures the discovery of target-specific aptamers because target specificity is inherently incorporated into the aptamer design phases. Hence, SCIA will significantly enhance aptamer discovery research relevant to dealing with aptamer-based drug discovery for both therapeutic and diagnostic purposes, such as cases where PS is a target biomolecule. Here, our breakthrough achievements are as follows:

• An aptamer designing method involving SCIs among charges in a drug-target biomolecule complex to help select ABBs
• Nucleic acid aptamers for binding specifically to PS
• Aptamer-based therapeutic and diagnostic templates for diseases where PS is a target biomolecule

## Highlights

• Aptamer designing method involving screened Coulomb interactions among charges in a drug-target biomolecule complex
• Designing nucleic acid aptamers for binding specifically to phosphatidylserine
• Creating aptamer-based therapeutic and diagnostic templates for various diseases

## Graphical Abstract

**Figure:**
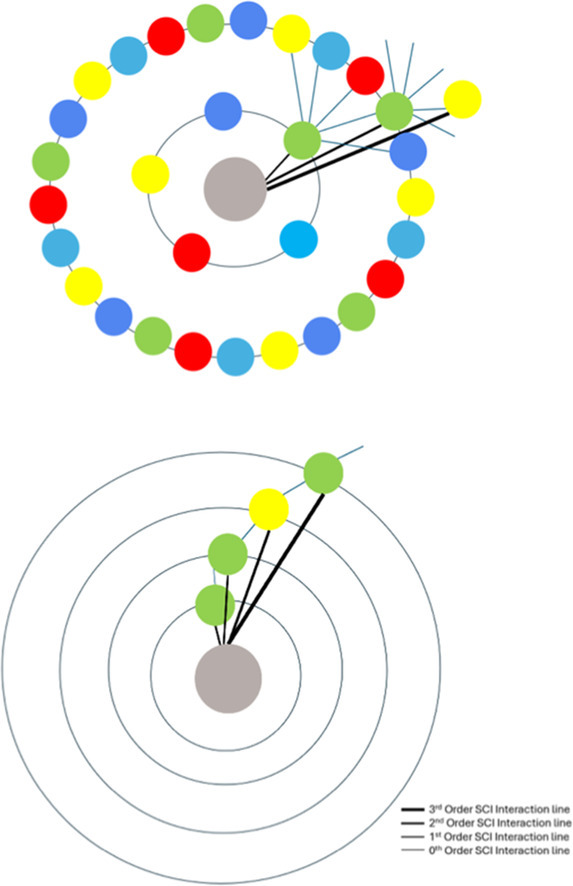

## Introduction

During a quarter of a century of aptamer research, starting with the discovery of systematic evolution of ligands by exponential enrichment (SELEX) [1,2], tremendous progress has already been made in both finding novel aptamer designing techniques and discovering nucleic acid aptamers with varied sequences for specific biomedical purposes [3]. We have substantial knowledge of the distinguishable features of aptamer design studies in which proteins, antibiotics, organophosphates, low-molecular-weight compounds, etc., are used as the drug targets [4]. Cell-specific aptamer recognition and uptake mechanisms, including formalisms behind the aptamer delivery, are also to some extent known to us [[5], [6], [7]]. Aptamer structure alteration may modulate aptamers’ interaction potency with target proteins, affecting related cellular behaviors, hence leading to the creation of templates for drug discovery in specific diseases [8]. In aptamer development, there are numerous strategies to overcome limitations related to designing a library, SELEX methodologies, and chemical modifications in nucleic acids for achieving enhanced applicability in therapeutics, diagnostics, drug delivery systems, and molecular imaging [9].

Despite huge progress, we are yet to achieve concrete advancement in many areas especially in the following two: (i) how we may select aptamer building blocks (ABBs), which are nucleotides adenine (A), guanine (G), cytosine (C), thymine (T) or/and uracil (U) to create nucleic acid aptamers (NAAs) by considering especially the physicochemical properties of both ABBs and target biomolecules, and (ii) how we may understand aptamer interactions with target biomolecules through analytical and experimental quantification of the vital statistical mechanical entity, the energetics of biomolecular association/dissociation revealing the strength of binding phenomenon. We aimed to develop an energy-based method, a screened Coulomb interaction approach (SCIA) that is capable of addressing both of these issues [10]. As aptamers are expected to be target-specific agents, we consider here using a phospholipid as a biomolecule target that participates in both constructing cellular membranes [11] and engaging in vital cellular signaling pathways [12]. Our main objective here is to deduce interaction energies (e.g., binding energy, *E*_SCI_) considering a screened Coulomb interaction (SCI) formalism, which are associated with aptamer-target biomolecule association (see “*derivation of binding energy, E*_SCI_” in Supp. Mat.) [10,11,13]. It will help in a reverse manner by providing energetic parameters that are important in selecting optimal aptamers for the specific target biomolecule.

Various types of phospholipids play vital roles in cellular membrane structure and function. In eukaryotic cell membranes, the major structural phospholipid components are the glycerophospholipids phosphatidylcholine (PC), phosphatidylethanolamine (PE), phosphatidylserine (PS), phosphatidylinositol (PI), and phosphatidic acid (PA) [14]. PC comprises more than 50% of the phospholipids and functions as a neutral lipid, exerting no overall charge and no intrinsic curvature, which is partly responsible for creating membrane planar bilayers. Research on PC in therapeutic and diagnostic drug discovery has emerged as a critical area of inquiry due to its fundamental role in cell membrane structure and function [11,13], as well as its involvement in various diseases, including cancer, inflammatory bowel disease, and neurodegenerative disorders [15,16]. We have designed a set of aptamers for PC, applying SCIA [17]. As we also succeeded in designing aptamers generally for various types of biomolecules like lipids, e.g., ceramide (US patent pending), and proteins, e.g., Bcl-2 (US patent pending), we applied the same SCIA for designing aptamers for PS, which is a vital target in drug discovery, especially including the purposes of designing aptamers (see Table 1). In Table 1, the collected studies highlight the role of PS in therapeutic and diagnostic drug discovery, where specific agents, including NAAs, may play crucial roles in drug discovery. A few of our own (by Ashrafuzzaman and colleagues) studies have already made some considerable progress in creating the background, particularly in the designing of NAAs capable of detecting specific target molecules like PS [[18], [19], [20]], which is a vital lipid that participates in maintaining the normal molecular biology of cells. Moreover, our developed theoretical strategies demonstrate that biomolecules like aptamers can specifically bind with lipids through electrostatic charge-charge interactions, including considering screening effects [21], helping them to experience improved liposomal binding stability, cellular uptake, and target-specific therapeutic delivery [20].

**Table 1:** PS is a target in biology for therapeutic and diagnostic drug discovery.

Disease/application	Available aptamer	Research phase	Application (drug discovery)	Method of design	Reference
Apoptosis/tumor cell.	AAAGAC and TAAAGA	preclinical	Diagnostics	EFBA (computational seed-and-grow)	[[18], [19], [20]]
Circulating tumor-derived exosomes (cancer biomarker)	No aptamer	Preclinical	Diagnostics	ELISA-style capture/detection using PS binding reagents	[22]
Cancer immunotherapy / tumor targeting (antibody)	Bavituximab (PS-targeting monoclonal Ab)	Clinical (phase I–III)	Therapeutics	Antibody development / conjugates; designed to bind externalized PS	[23]
PS-binding peptides & small molecules	Peptide ligands	Preclinical / in vitro	Therapeutics	Peptide design and screening; plate-based liposome binding assays, in silico docking and experimental validation	[24]

The redistribution of PS from inner (scoring around 10% of the membrane lipids) to outer plasma membrane is referred to as PS externalization and is an early marker of apoptosis [[25], [26], [27]]. The presence of PS may alter aggregation pathways and increase amyloid aggregation in TSEs [28]. The amyloid aggregation helps trigger apoptosis, leading to neuronal cell death [29]. Annexin V (annexin A5), a naturally occurring human PS-binding protein, has emerged as either a radionuclide-containing or a fluorescent probe to detect PS [25]. However, annexins exhibit disadvantages, including high uptake in normal tissues, long half-life in non-target tissues, high radiation burden with radiolabeled tracers, and laborious issues around labeling [30]. Therefore, new agents, such as aptamers, may create an alternative class of chemicals that may help detect PS [18,19] in disease to aid in the diagnosis of specific pathological aspects.

Research targeting PS has expanded across both diagnostic and therapeutic drug discovery avenues, reflecting its important roles in apoptotic and malignant cells. In the diagnostic aspect, Ashrafuzzaman and colleagues first used an entropic fragment-based approach (EFBA), including utilizing a computational seed-and-grow design strategy to generate the DNA aptamers and succeeded in finalizing a few NAAs like AAAGAC and TAAAGA, which exhibited selective affinity for PS in preclinical in-vitro assays, highlighting their potential as molecular probes for apoptosis-based tumor detection by quantifying PS externalization across the plasma membrane’s specific apoptotic phase induced by chemotherapy [[18], [19], [20]]. Sharma and colleagues demonstrated that PS-positive circulating exosomes can serve as early biomarkers of malignancy through an ELISA-based detection platform, establishing PS as a clinically meaningful indicator even in the absence of nucleic acid ligands [22]. Therapeutically, PS has been leveraged as a target for immune modulation, exemplified by the monoclonal antibody bavituximab, which was evaluated in phase I–III clinical trials for non-small-cell lung cancer and designed to bind externalized PS on tumor vasculature [23]. Further preclinical work has explored PS-binding peptides, with Kapty and colleagues identifying peptide ligands through iterative *in silico* modeling and liposome-binding assays, supporting their application as targeted delivery of pro-apoptotic agents [24]. Collectively, these studies underscore the translational relevance of PS as a useful molecular target and reveal multiple modality-specific strategies for exploiting PS exposure in cancer diagnostics and therapeutics.

Despite advances, challenges remain in fully exploiting PS’s potential in drug discovery. The translation of lipid-targeted agents into clinical practice is limited [31], and designing an aptamer to target PS might be challenging in terms of specificity. No aptamers that directly bind with PS with validated high specificity have been published, except for a few (designed using EFBA) studied by Ashrafuzzaman and colleagues [18,19], where specific aptamers were shown to have preferred membrane binding when PS was present in liposomes predominantly made of PC. The key is that the membrane presence of a modest amount of PS drives many of its specific aptamers to bind with it, validated in both liposome and cell assay studies [[18], [19], [20]]. In this article, we shall revise our strategies to design aptamers [18,19], specifically for PS, where we have pre-analyzed the physicochemical properties of ABBs and PS to deduce physicochemical parameters, such as density of interacting charges and their average separation (we call it ‘lattice constant’) as they approach each other to establish physical interactions that may be qualified by interaction energetics [10]. To construct an aptamer, we need to create a chain of ABBs, so we may apply a one-dimensional (1D) approach describing aptamer length (*l*_Apt_) and the aptamer’s linear interactions with PS, considering their charges and dimensions (see for ABBs, ref [32] and PS, refs [33,34]. Although we can’t deny the possibility of an aptamer to laterally interact with target biomolecules [10], we are limiting our analysis here, for simplistically addressing the rather complicated energetics of biomolecular interactions, to a linear aptamer-PS interaction scenario.

***One-dimensional modeling of a chain of ABBs.*** In 1D modeling of a chain of ABBs, we may apply some conceptual approaches and approximations. If we see the growth of aptamer length as we keep adding ABBs, we let the aptamer grow as a 1D-like rod. As our aptamers are not too long, they maintain 1D rod-like structures. As these small-length aptamers approach lipid (especially head group region), we may likely approximate the charge distribution in 1D without much compromise as long as it’s an interaction between a single aptamer and a single lipid, producing a scalar entity, binding energy. However, as aptamer length increases, we may see many lipids being covered within the interparticle interacting regime; hence, higher-order dimensional approaches may be required. So, for this case, the 1D approach is safe. While considering the dimensions (∼cross-sectional lengths) of nucleotides, let us just consider classifying all 5 nucleotides into two groups, namely purine (Pu): A, G, and pyrimidine (Py): C, T, U with identical linear (cross-sectional) dimension *a*_Pu_ and *a*_Py_, respectively, due to their structural differences, consisting of two rings and single ring, respectively. Firstly, two-ring versus one-ring architecture makes purines bulkier in three-dimensional (3D) shape and electron distribution [35]. Secondly, from the functional/structural perspectives, the overall base-pair width is constant in B-DNA because A–T and G–C base pairs pair a purine with a pyrimidine. But purines show larger groove-occupancy and different opening energetics compared with pyrimidines [36]. Watson and Crick established the fundamental geometry of B-DNA, describing complementary base pairing, approximately ten base pairs per helical turn, and an axial rise of about 3.4 Å (0.34 nm) per base pair [32]. Based on this, we defined an axial size for each nucleobase as a 1D modeling parameter, assuming each purine contributing approximately (*a_Pu_*=) 0.34 nm and each pyrimidine about (*a_Py_*=) 0.30 nm (just a judicious choice) along the DNA axis, hence our assumption stands at *a_Py_* ≈ 0.9*a_Pu_*. In recent studies, the stacking correlation length in ssDNA has been reported using the modern single-molecule/stacking analyses, quoting that per-base contour/stacking lengths are 0.33–0.39 nm, depending on sequence and conditions [37]. All these help us propose a 1D modelling, which will help us provide measures of important lattice constants (see “*1D Modeling of Charge Distribution in PS-ABBs Complex*” in Supp. Mat.) as well as the length of any aptamer *l*_Apt_, following a simple formula, as follows:

(1) $$l_{\text{Apt}}=n_{\text{Pu}}\times a_{\text{Pu}}+n_{\text{Py}}\times a_{\text{Py}}$$

where the respective aptamer consists of *n*_Pu_ and *n*_Py_ number of purines and pyrimidines, respectively. Hence, the total number of ABBs (*n*_ABB_) in any aptamer, *n*_ABB_ = *n*_Pu_+ *n*_Py_.

The functionality of aptamers may depend on both the sequence (choice) of ABBs and *l*_Apt_, hence these are essential parameters in our SCIA-based design of aptamers [10]. In SCIA, as an aptamer or even an ABB approaches a target biomolecule, we equivalently consider the complex to consist of a many-charge system [10], in which charges interact with each other following a SCI formalism in condensed matter phase, first developed by Ashrafuzzaman and colleagues [21], then elaborated to address the membrane interactions of ion channels [11,13]. This approach helps us deduce the energetics (especially, the free energy of charge-charge association/dissociation, ΔG) of PS-ABB interactions for various (interaction) screening orders (see “*derivation of binding energy, E*_SCI_” in Supp. Mat.)), hence helping us select appropriate aptamers for PS.

## Methods and Materials

Three different methods have been applied. All of them will be briefed here, including the required materials.

### Analyzing physicochemical properties of PS and ABBs

In SCIA, we need to understand the functional charges the participating biomolecules carry in aqueous phases [10]. Here, we analyzed the functional sites in PS and ABBs, understanding the distribution of electronic charges, which helps us find two parameters, namely, effective charges that would participate in SCIA in a PS-ABB or aptamer complex, and lattice constants. Lattice constant is the average intercharge separation, obtained by relaxing the charge particles’ status to freely distribute along a 1D model distribution (approximated here for making our calculation easier) to achieve an equilibrium distribution of charges. Details are presented in the Supp. Mat. (see ‘*1D Modeling of Charge Distribution in PS-ABBs Complex*’).

### Numerical computations to address the SCI energetics of drug-target interactions

We treat the interaction between a drug and a target biomolecule in the aqueous phase as a combination of interactions among charges in a many-charge system, following our previously developed theoretical SCI template, referring to interactions among particles (or charges) of a many-body system in a condensed matter phase [20]. This theory was revised for similar interactions among biomolecules in an aqueous phase in refs [11,13]. The detailed analyses are presented in the Supp. Mat. (see ‘*Derivation of binding energy, E*_SCI_’), which presents a strategic analysis on how to calculate the binding energy, *E*_SCI_, among charges under SCI interactions. It is to be noted that to calculate the values of *E*_SCI_ for various SCI orders through numerical computations (NCs), we have to apply a Fourier transformation to our theory using our own developed algorithm in Mathematica 9 (https://www.wolfram.com/).

### *In vitro* aptamer-liposome binding assays

Liposomes were constructed using 1,2-dipalmitoyl-sn-glycero-3-phosphatidylcholine (PC) and 1,2-dipalmitoyl-sn-glycero-3-phosphatidylserine (PS). Lipids and aptamers were supplied by Avanti Polar Lipids (700 Industrial Park Drive Alabaster, Alabama 35007-9105) and HVD Biotech (Vertriebs GmbH., Wurzbachgasse 18, 1150 Vienna, Austria), respectively. Standard binding assays were utilized (for details, see previous publications from our group, e.g., refs [18,19]) to separate liposome-bound aptamers from unbound ones in buffer (HEPES). Aptamers were added from a primary 1 mM stock solution in Tris/EDTA buffer.

The solution with PS-bound aptamers was then investigated for aptamer quantification. DDM was developed and applied to detect the lipid-bound aptamers in mole (M) fraction in pure PC or PC and PS (10% PS incorporated in predominantly PC liposomes) liposome systems using absorbance spectroscopy [38,39]. We used a NanoDrop (from ThermoFisher Scientific) to get the absorbance spectra specific to the aptamers. The wavelength, λ_DNA_ =260 nm, for the DNA aptamer detection, was set (see Sigma-Aldrich manual). We performed the spectroscopy and quantified the aptamer concentrations in liposome-bound conditions [38]. From their detected concentrations, we calculated molarities of the lipid-bound aptamers in the incubation tube. For additional details on how the technique works, see ref [39].

## Results

We have three independent sets of results derived from three distinguished types of research. They are presented here in independent subsections.

### Analyzing physicochemical properties of PS and ABBs

**Fig. 1:**
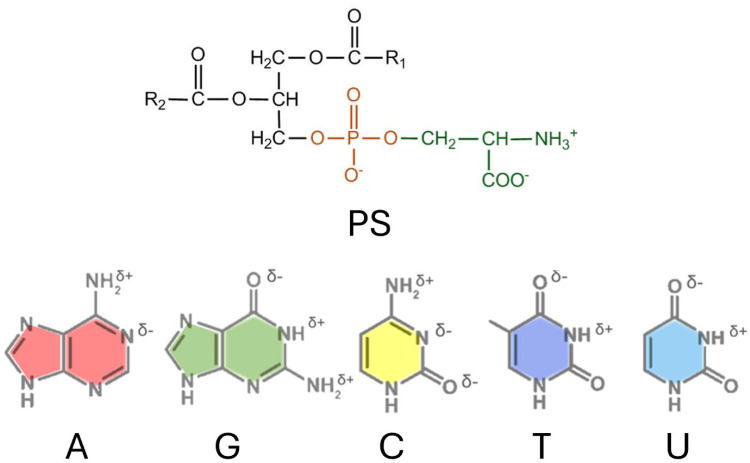
Structure with charge distributions of PS (top panel) and ABBs (bottom panel) [10]. The assigned color coding, A (red), G (green), C (yellow), T (blue), and U (light blue), will be maintained throughout the article.

Before performing NCs, PS and ABBs were analyzed to determine their functional groups participating in biomolecular interactions in aqueous phases. PS is a net negatively-charged phospholipid; for charge distribution, see Fig. 1, at a neutral pH. The functional groups and estimated charges of PS and all ABBs are presented in Table 2.

**Table 2:** Net charge amount and charge sites in PS and nucleotides.

Biomolecule	Charge Side	Net Charge	Total Charge
PS	Po_4_^−^,NH_3_^+^,CO_2_^−^	-e	Negative (-)
A	Hδ+, Nδ-, PO_4_^−^	-e	Negative (-)
G	Hδ+, Hδ+, Oδ-, PO_4_^−^	-e, δe	Negative (-)
T	Hδ+, Oδ-, PO_4_^−^	-e	Negative (-)
C	Hδ+, Nδ-, Oδ-, PO_4_^−^	-e, δe	Negative (-)
U	H δ+, Oδ-, PO_4_^−^	-e	Negative (-)

PS preferred ABBs at different SCI orders are selected from A, G, C, and T for designing DNA aptamers, or A, G, C, and U for designing RNA aptamers. An important observation here is that both C and G carry four functional charges, while A, T, and U carry three functional charges (see Table 2), placing them in two charge distribution classes [10].

### NCs addressing the interaction energetics of PS with an ABB

**Fig. 2:**
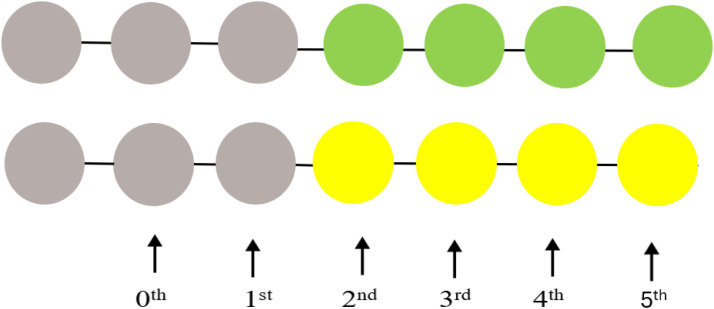
1D model structure of intracharge distribution for direct PS-ABB SCI. PS has three charges (leftmost, dark-brown), G (green) has 4 charges, and C (yellow) has 4 charges (see Fig. 1). The lengths are different, considering the difference in their dimensions (explained in Fig. Supp. 2). The leftmost particle (charge) interacts with subsequent charges following 0^th^, 1^st^, 2^nd^, 3^rd^, and 4^th^, and 5^th^ order SCI formalism. Hence, PS-ABB interaction is switched on from the 2^nd^ order SCI and beyond. G and C are chosen randomly to explain the modeling and the difference in the lengths of the PS-ABB pair, as ABBs have been assumed to show modestly different lengths, considering their purine or pyrimidine classifications (details in Fig. Supp. 2).

**Fig. 3:**
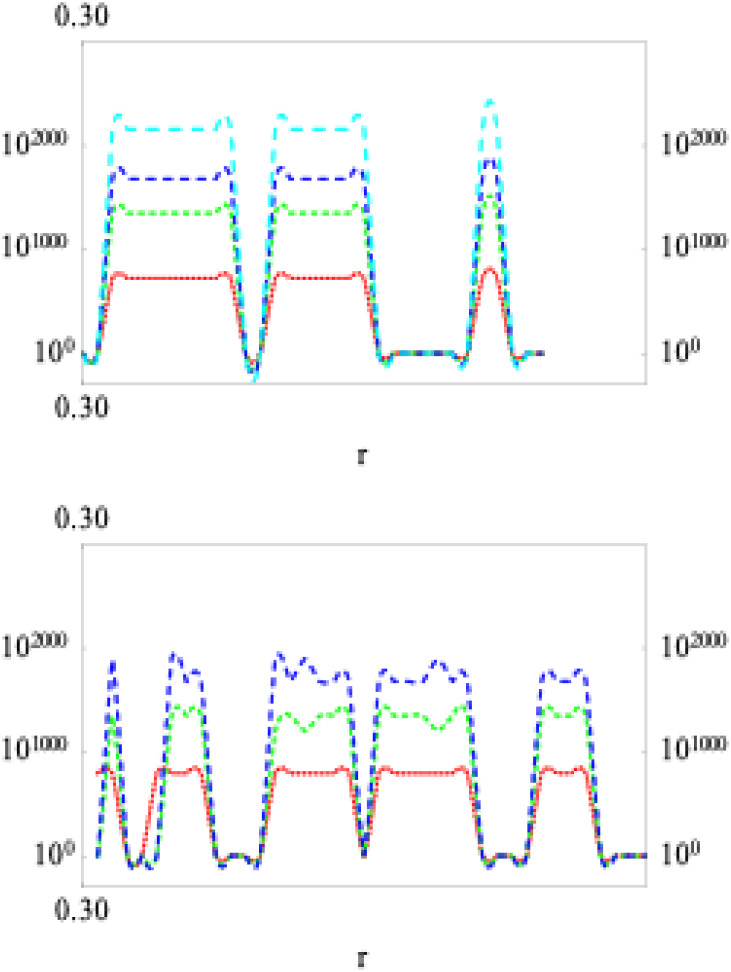
E_SCI_^0^ graph for PS interacting with C (top panel), with A (bottom panel). Lines are for SCI or i= 1, 2, 3, and 4 (bottom to top). C and A represent two charge groups in nucleotides with 4 and 3 charges (Table 2), respectively. In performing NCs, lattice constants, a_0_ corresponding to ABBs, are chosen from Table Supp. 1. We present data for PS interactions with C and A, which represent two classes of ABBs, namely those with different dimensions and functional charges. Lattice constants are taken from Table Supp. 1 in NCs.

In this study, we wished to design an aptamer with up to 10 ABBs. Mathematica 9 algorithms were applied to perform NCs, considering the Fourier transformation on the interaction potentials [10,11,13]. We first select the zeroth (*i=0*) order SCI energy, *E*_SCI_^0^, following an identical strategy as explained in the supplementary materials (see *Derivation of binding energy, E*_SCI_). Here, *E*_SCI_^0^ is the value of *E*_SCI_ at the 0^th^ order SCI (*SCI*^0^). We would then continue to select the subsequent ABBs for all other *SCI*^i^, with *i*=1, 2, 3,…., etc., (see section 3.3) based on free binding energetics [10,11,13,21]. For each value of *i*, we estimated the values of the highest and lowest points at binding energies, equivalent to their universal trends (see Fig. Supp. 1). For the intracharge distribution modeling in a PS-ABB pair (see Fig. 2), the NC-deduced data for various SCI order energies (within the spectrum of *E*_SCI_^0^) are plotted in Fig. 3. This helps determine energetics of association/dissociation, whose related energy values are presented in Table 3. For ABB selection, the actual energy scaling isn’t required. However, interested researchers may deduce it using the detailed explanations and derivation protocols provided in ref [13]. We note that in 0^th^ order *E*_SCI_ calculations, we can apply our generalized SCIA as the intracharge distribution allows us to consider the interactions following SCIA formalism [10], where we can consider charge distribution following 0^th^, 1^st^, 2^nd^, 3^rd^,…, etc. SCIs (Fig. 2). The PS-ABB complex is nothing but a complex of many (mutually polarizable) functional charges, instead of merely two overall charges that could represent the participating two biomolecules, hence would be equivalent to a direct Coulomb interaction (DCI). Hence, a direct PS-ABB complex considers intracharge distribution to determine the energy for *SCI*^0^ using SCIA.

**Table 3:** Matrix of Log values of energies (Log *G*_I_ (High) and Log *G*_II_ (Low)) at every SCI order (within the framework of calculating *E_SCI_^0^*) for SCI orders (1≤ *i* ≤5) within any direct PS-ABB complex. *G*_I_ and *G*_II_ are values of the energy at two extreme ends of its transitions (e.g., see Fig. 3). Compared to higher-energy values, the lower energy values are negligible for all SCI orders. Hence, consideration of the 2^nd^ column values (Low) is irrelevant, and the ABB selection can be made based on high energy values (High) only. High and Low values of energies are chosen at the two terminals at every energetic transition. Lattice constants are taken from Table Supp. 1 [32,40,41] in performing NCs.

Variation in aptamer sequence can occur when more than one ABB corresponds to the same lowest Log ΔG (≈Log *G*_I_) value for the same SCI order (see Supp. Mat.: ‘*Derivation of binding energy, E_SCI_*’). However, in this case, the 0^th^ order unambiguously shows only G to appear systematically (1^st^ and 5^th^ order in Table 3) with the lowest Log ΔG values.

### NCs addressing the interaction energetics of PS with a chain of ABBs

**Fig. 4:**
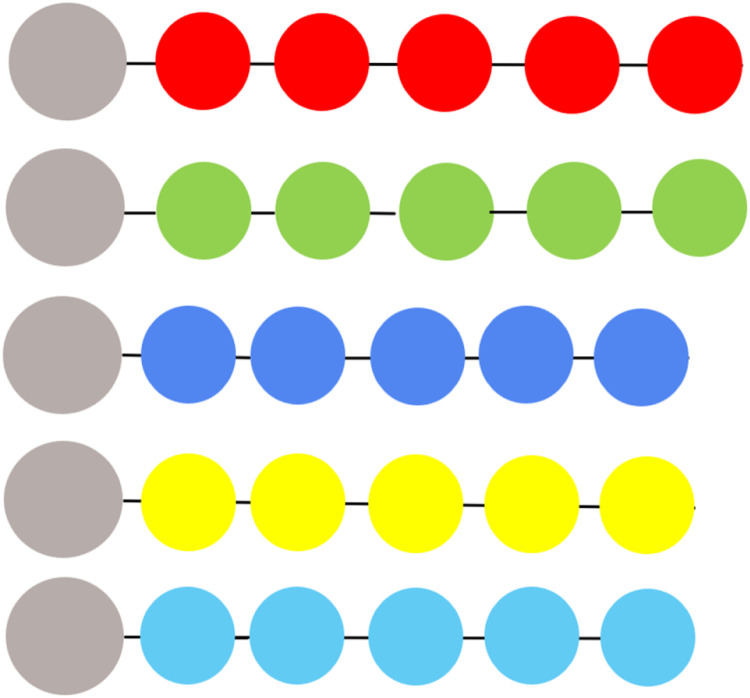
A 1D model structure of the interaction of PS (leftmost brown sphere) with poly-purine or poly-pyrimidine aptamers (SCI interactions) (only up to i=4, i.e., 0^th^, 1^st^, 2^nd^, 3^rd^, and 4^th^ order SCI scenarios have been sketched for representation purposes). The top two linear arrangements represent a PS interaction with a chain of purines (A: red, G: green), the third to fifth arrangements represent a PS interaction with chains of pyrimidines (T: blue, C: yellow, U: light blue).

NCs are performed for the scenario in Fig. 4 for all SCI orders of choice. 0^th^ order SCI interaction energies have already been deducted in Table 3. Table 4 presents data for SCI orders *i*= 1-9. All High values represent Log *G*_I_, hence Log ΔG (≈Log *G*_I_), due to the same analogy, explained in the caption of Table 3.

**Table 4:** Matrix of Log values of energies (Log *G*_I_ (High) and Log *G*_II_ (Low)) at every SCI order for *i*= 1-9. Lattice constants are taken from Table Supp. 2 in NCs. Coulomb interaction (CI) (i= 0) refers to the analysis of energy values presented in Table 3.

### Designing aptamers and selecting the best candidate(s)

**Fig. 5:**
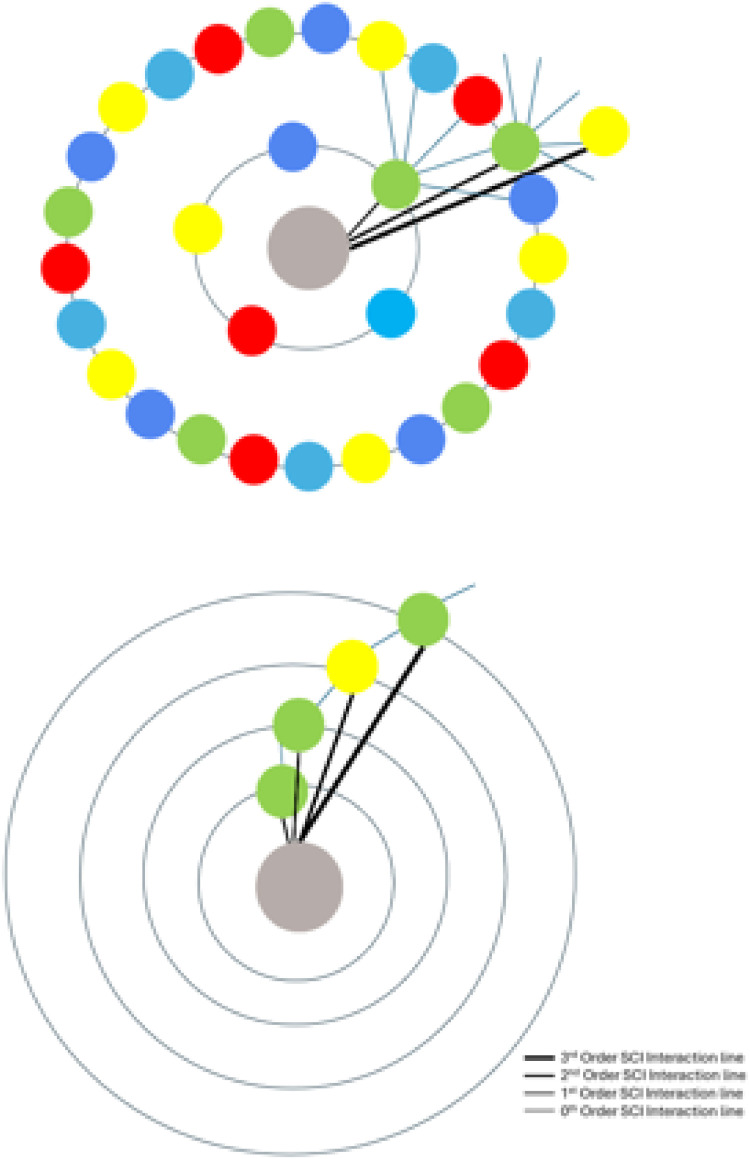
Possible number of SCIA-designed aptamers compared to their maximum possibility. Lower panel: best-predicted ABBs in each order/position generated by SCIA (sketched here just up to SCI order 3). Upper panel: the shell model representation of the theoretical possibilities of choosing ABBs at every SCI order. For 0^th^, 1^st^, 2^nd^, …, etc., SCI order, there can be 5, 25, 125, …etc., number of ABBs accommodated (see Supp. Mat.: “Shell model to theoretically demonstrate target interaction of ABBs leading to estimating possibility of aptamer design”) [10].

The data in Table 3, Table 4 suggest that the options of the PS aptamer (PSApt) come from a matrix of ABBs, as follows:

[G G C G G C G (A or G or C or T or U) (A or T or U) G], which can be diagrammatically presented as in Fig. 5 (lower panel). To be noted here that at the leftmost position, which would qualify to perform direct Coulomb interactions (DCIs) with PS, we already discovered G as the candidate ABBs (see Table 3). The lower panel of Fig. 5 demonstrates the number of options applying our SCIA, which we could fit in our proposed shell model, see Fig. 5 (upper panel). Compared to the theoretical maximum possibility (see Supp. Mat.: “Shell model to theoretically demonstrate target interaction of ABBs, leading to estimating possibility of aptamer design”), our SCIA has been able to reduce the number of ABBs at every SCI order, hence the number of aptamers, substantially, e.g., for aptamers with 10 ABBs to target PS, only to 13 (see Table 5). Hence, the shell (in Fig. 5, lower panel) looks cleaner with only a few ABBs compared to the theoretically ABB-crowded shell (in Fig. 5, upper panel) [10].

**Table 5:** The SCIA predicted PS aptamer sequences (PSApt 1-13).

Universal aptamer		DNA aptamer		RNA aptamer	
PSApt 1	GGCGGCGAAG	PSApt 4	GGCGGCGTAG	PSApt 9	GGCGGCGUAG
PSApt 2	GGCGGCGGAG	PSApt 5	GGCGGCGTTG	PSApt 10	GGCGGCGUUG
PSApt 3	GGCGGCGCAG	PSApt 6	GGCGGCGATG	PSApt 11	GGCGGCGAUG
		PSApt 7	GGCGGCGGTG	PSApt 12	GGCGGCGGUG
		PSApt 8	GGCGGCGCTG	PSApt 13	GGCGGCGCUG

All aptamers (see Table 5) can be classified into the following three categories:

– Universal aptamers (non-nucleic acid type-specific): PSApt 1-3, without T and U
– DNA aptamers: PSApt 4-8 with T, and
– RNA aptamers: PSApt 9-13 with U.

While designing PC aptamers using identical SCIA, in addition to these three above categories of aptamers, like those discovered for PS, we found another category, ‘universal aptamers (hybrid)’, which consisted of both T and U in all sequences [17].

**Fig. 6:**
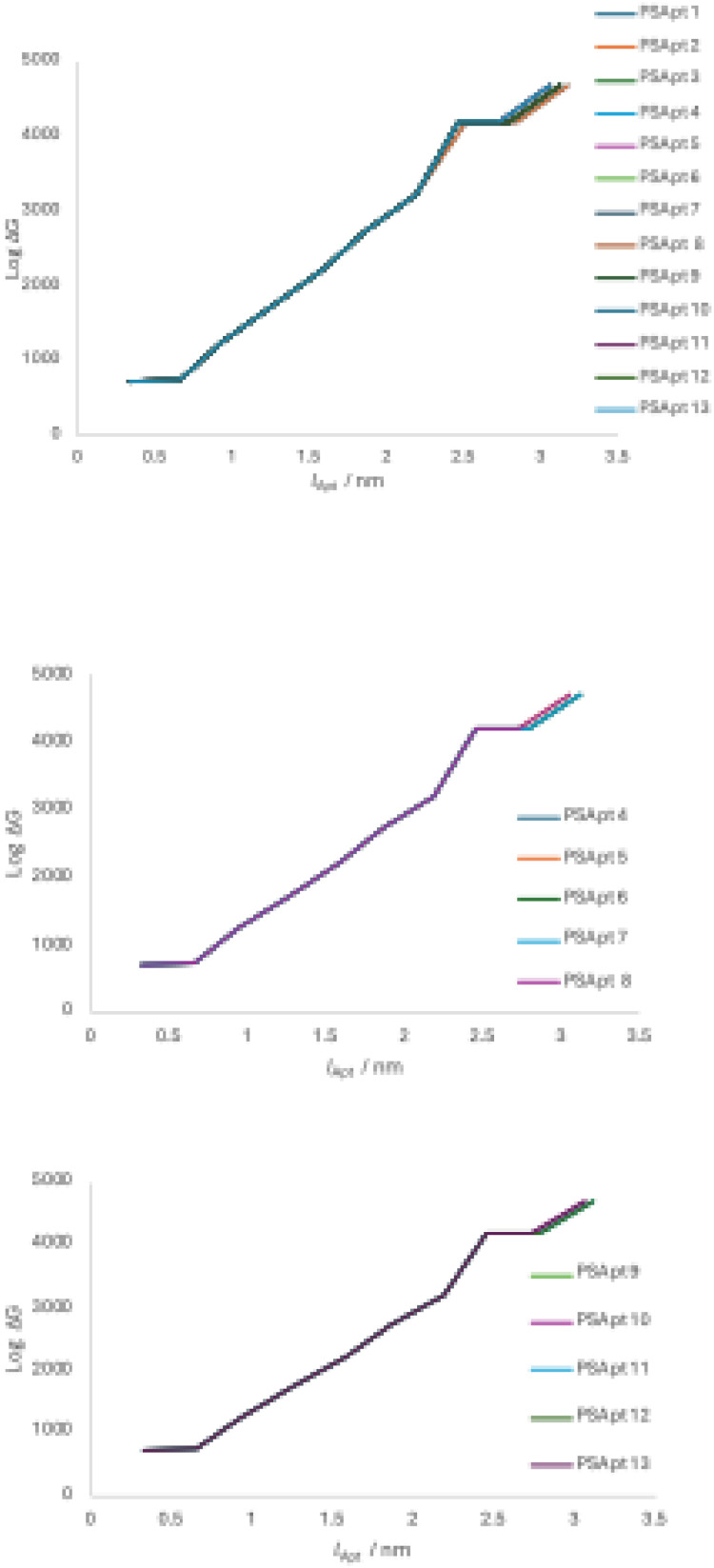
Plot of Log of the free energy as a function of *l*_Apt_ (all aptamers from Table 5) (top panel). The middle and bottom panels plot the DNA and RNA aptamer cases separately.

Table 5 presents us with a still huge number of aptamers, considering that we wish to pursue their *in vitro* target binding experimental validation, including, especially, cytotoxicity assay studies [20]. Therefore, we developed an analytical method that would help us further reduce the number and find only a few as the best aptamers as candidate drug agents. While considering the dimensions (∼cross-sectional length) of nucleotides, as explained earlier, we consider *a*_Pu_ and *a*_Py_ to be the cross-sectional dimensions (diameters) of any purine and any pyrimidine, respectively. Considering Eq. (1), we measured the values of *l*_Apt_ for all 13 aptamers (Table 5). The respective values of log ΔG (Table 4) are plotted against the corresponding *l*_Apt_ covering different SCI orders (see Fig. 6).

**Fig. 7:**
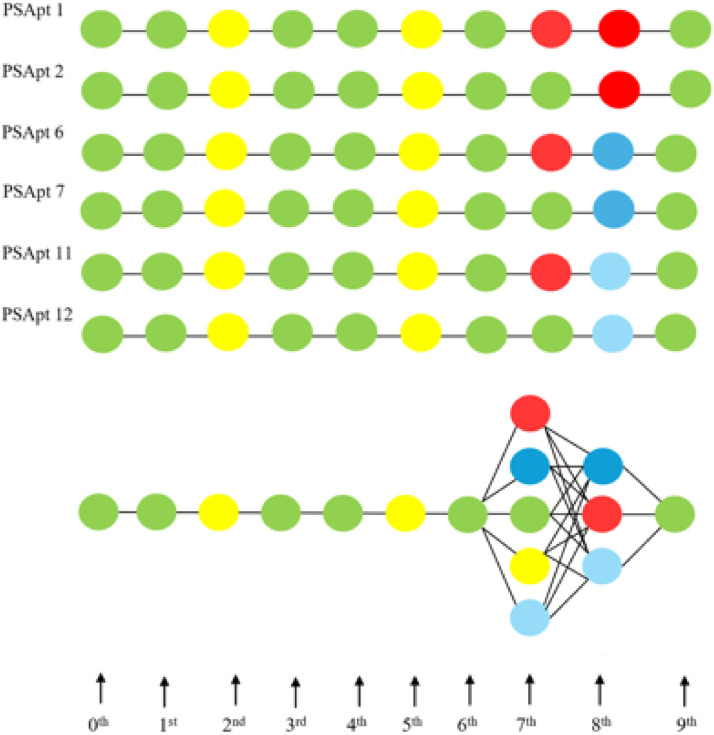
Finalized PS aptamers (PSApt 1, 2, 6, 7, 11, 12) with their distinguishable type (nucleic acid specificity), lengths, and variation in ABBs. The bottom panel represents all possible 13 aptamer options (Table 5). Our analyses in Fig. 6 have helped us reduce the number by more than half, and with varied lengths (PSApt 1, 2, 6, 7, 11, 12).

Both PSApt 1 (GGCGGCGAAG) and PSApt 2 (GGCGGCGGAG) (see top panel) have the same lowest energy per length, suggesting that these two could be the best overall aptamer templates for PS. Both GGCGGCGAAG and GGCGGCGGAG, both GGCGGCGATG and GGCGGCGCTG (middle panel, Fig. 6), and both GGCGGCGAUG and GGCGGCGGUG (lower panel, Fig. 6) have the lowest energy per length in the three categories, universal aptamer, DNA aptamer, and RNA aptamer, respectively, suggesting that these 6 aptamers could be the best templates in their respective categories for binding with PS, which is a considerable reduction in number from all possible 13 (see bottom panel of Fig. 7). Although they vary in their lengths (Fig. 7), only beyond SCI order 7^th^ they show both nucleic acid specificity and associated aptamer length variation. Hence, to obtain nucleic acid specificity with distinguishable target binding energetics in our aptamers, we must consider designing them with more than 8 nucleotides in their ABB sequences. Therefore, for every target biomolecule, a certain minimum length of aptamers has to be judiciously chosen. Although we chose to design aptamers with 10 nucleotides, we could continue extending aptamer lengths, but performing NCs becomes too time-consuming, or even the computing power of the device may need to be upgraded.

### *In vitro* aptamer-liposome binding assays validating aptamers’ PS binding potency

**Fig. 8:**
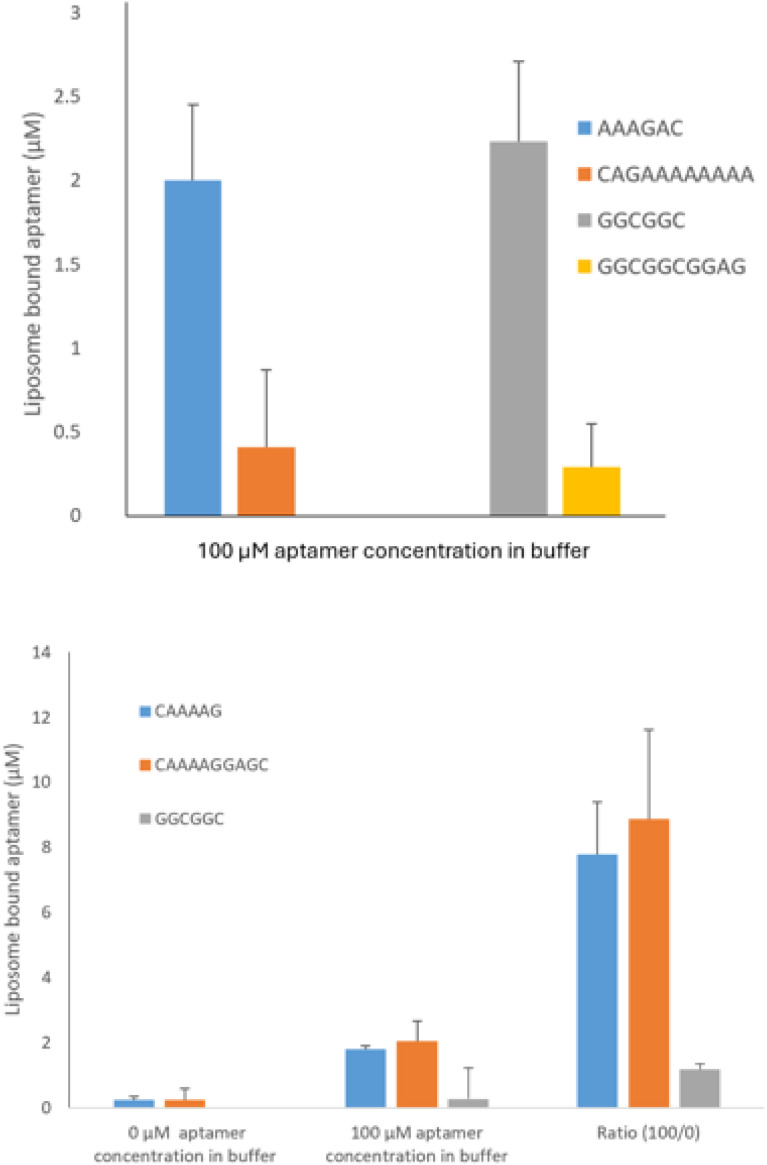
Liposome-bound aptamer concentrations (DDM detected) versus aptamer concentrations in liposome-bathing buffer. **Top panel**: we selected two sets of PS aptamers, GGCGGC, and GGCGGCGGAG (SCIA-designed), and AAAGAC and CAGAAAAAAAA (EFBA-designed). Varied sequences and lengths are considered to understand the relative binding potencies that might rely on both or either of the aptamer sequences and length. Liposome: 10% PS in predominantly PC liposome. **Bottom panel** [17]**:** we selected two PC aptamers (varied lengths), CAAAAGGAGC and CAAAAG (both SCIA-designed), mainly to compare with other biomolecule (e.g., PS here) targeted aptamers containing the same number of ABBs [18,19] regarding their relative liposome binding potencies. For the negative control (from the target lipid perspective), we used one of our PS aptamers, GGCGGC (SCIA-designed) [10]. Liposome (PC liposome): 0% PS in predominantly PC liposome.

Fig. 8 presents the plots of data from our selected aptamers’ *in vitro* liposome binding potency. We have selected a PS aptamer, GGCGGC, and its longer form GGCGGCGGAG (see Table 5, PSApt 2), mainly to compare if aptamer length has any effect that might be exerted while binding with the target biomolecule PS, besides understanding their general liposome binding potency [18,19]. We compared these data with data produced from identical experiments, considering two aptamers, AAAGAC and CAGAAAAAAAA, that we designed earlier using another method, EFBA [18,19]. These comparable data are presented in Fig. 8 (top panel). PS is found to favor short aptamers over long ones for considerable binding, irrespective of the techniques we utilized while designing aptamers.

In the bottom panel of Fig. 8, we present the plots of *in vitro* liposome binding potency data for the SCIA-method designed selected PC binding aptamer, CAAAAGGAGC, and its shorter form CAAAAG [17], mainly to compare with PS-targeted aptamers’ liposome binding potency containing the same number of ABBs, hence having corresponding identical lengths [18,19]. For the negative control (from the target lipid perspective), we used one of our SCIA-designed PS aptamers, GGCGGC [10]. The relative quantification of the liposome binding assay of this PS aptamer with PC aptamers will ensure whether our SCIA is capable of designing aptamers specifically for any biomolecule.

From previous studies [19,39], we found that at around the aptamer concentration of 100 μM, the binding properties may be inspected to compare between different sequences and also regarding target specificity. It’s a concentration at which the dose-response curve appears sensitive and changes (before running towards reaching the equilibrium or saturation phase) as a result of target binding. We followed it here and tested all 7 aptamers for their target binding potency at 100 μM.

As an exemplary case, we considered a DNA molecule (telomere sequence) AGGGTT as a neutral aptamer to both PC and PS [10]. Hence, as a negative or neutral control, we performed liposome binding assays with it. For both 100% PC and 10% PS doped PC liposomes, we measured negligible liposome-bound concentrations of 0.27±0.16 μM and 0.25±0.19 μM, respectively, of AGGGTT for the case when 100 μM AGGGTT was incorporated into the liposome incubating buffer, exactly like the case for PC and PS aptamer binding assays, explained for Fig. 8. The negligible liposome binding data of AGGTT clearly suggests that we need to consider the physicochemical properties of target molecules right during the aptamer design phases [10] to ensure achieving considerable aptamer binding with targets.

The relative quantification of the liposome binding assay of this PS aptamer with PC aptamers will ensure whether our SCIA is capable of designing aptamers specifically for any biomolecule. Results in Fig. 8 show that while PC aptamers bind to PC liposomes considerably, PS aptamers fail to do so, as they exhibit negligible liposome-binding potency (bottom panel). The upper panel data clearly suggest that, irrespective of the methods utilized for designing aptamers, shorter aptamers are better PS binding than their longer peers. Similar results were reported by us (Ashrafuzzaman and colleagues) in our first set of publications with EFBA-designed PS aptamers more than a decade ago [18,19].

## Discussion

We developed an energy-based method to design aptamers specifically for a biomolecule. In this case, we chose PS as the aptamer target biomolecule due to its versatile roles associated with various diseases (e.g., see refs [[42], [43], [44], [45]]). Hence, designing PS-binding aptamers may lead to the discovery of therapeutic and/or diagnostic aptamer-templates with potential biomedical applications. Following our developed theoretical physics technique in condensed matter charge systems [21], we constructed an SCIA platform to consider interactions among many charges in a drug-target complex [11,13]. Incorporating Mathematica 9 algorithms into our NCs, we could address the PS-ABB interaction energetics for various SCI orders, which helped select the best ABB for every SCI order, considering its lowest energetic cost in interacting with PS. This way, with a preassumed length of aptamers (reflected in maximum SCI order), we could ensure obtaining aptamers that would bind with PS, considering the minimum aptamer-PS interaction energy. With the same minimum aptamer-PS interaction energy conformation, we obtain a set of aptamers, each of which would equally qualify energetically to bind with the target PS. Although it’s a considerable reduction in the number of options of aptamers from the maximum possibility (see Fig. 5), we would still end up having too many aptamer agents. Therefore, we aimed at resolving this issue utilizing another novel concept (pending US Patent) in which we thought to consider the energetic costs per length of aptamer, as not all aptamers get discovered with the same length, despite having the same number of ABBs, due to the reason that the dimension of an ABB may be nucleotide-type specific. This way, we could further reduce the number of aptamer candidates and find only a subset containing a few from the set of a large number of discovered aptamers. In our approaches, we also distinguished among the possibilities of finding nucleotide (RNA or DNA)-specific aptamers or nonspecific ones, or even combinations thereof (manuscript in review). This is an extension of our other concomitant studies with the same SCIA technique to design aptamers for two other vital lipids, PC [17] and outer mitochondrial membrane transport-regulating channel-forming ceramide [46] (pending US patent), and an antiapoptotic protein, Bcl-2 [47] (pending US patent). In the designing phases, we had to be careful in choosing specific parameters (see Supp Tables 1, 2), derived from the functional dimensions of target biomolecules and ABBs in respective physiological conditions [32,40,41]. SCIA may therefore appear as a universal platform, capable of designing aptamers for diversified biomolecular targets of specific medical importance.

The *in vitro* liposome binding assay demonstrated mainly two things, namely, our SCIA is capable of designing target biomolecule-specific aptamers, and that these aptamers, compared to other aptamers with different target biomolecule (e.g., PC) specificity [17], are quite potent when it comes to binding optimally with their target biomolecules. The aptamer length measurement and per length energy analysis-based finalization of aptamers (Fig. 7) were performed after we had already demonstrated our *in vitro* binding assays (Fig. 8). Therefore, we did not consider using specifically the finalized sequences in Fig. 7. However, we wish to do so later in our rigorous experimental assay studies, including aptamer-based cell cytotoxicity assays that we demonstrated recently [10].

Importantly, shorter PS aptamers have been universally found to bind better than longer ones with PS-containing liposomes. We know PS is found in the intracellular plasma membrane bilayer [12,48]. Hence, to bind with PS, aptamers need to penetrate the membrane and reach the intracellular bilayer leaflet. This process is relatively easier for shorter aptamers than their longer peers, whose relatively more complex and higher-order structures (tertiary/quaternary) [49] might hinder them from their expected membrane penetration and reaching out to the intracellular leaflet of the bilayer membrane where their target PS resides. This may also draw similar reasoning to small nanoparticles, which are more capable of perturbing membranes [50].

To convincingly demonstrate specificity, we may plan (in our future studies) on incorporating additional control experiments using alternative anionic lipids and employ quantitative techniques like surface plasmon resonance (SPR) [51] and/or microscale thermophoresis (MST) [52] to measure target-specific drug binding affinities and kinetics. SPR is a highly effective, label-free, and real-time analytical technique used to study the binding affinity, kinetics, and specificity of drugs (small molecules, proteins, peptides, aptamers) to target lipid membranes or specific lipids. MST is a powerful, immobilization-free, fluorescence-based technology used in drug discovery to quantify the binding affinity between small-molecule drugs and target lipids. It works by measuring the movement of molecules in a microscopic temperature gradient, which changes upon binding, making it highly sensitive to alterations in hydration shells, charge, or molecular size. There is another vital issue of aptamers’ high target affinity versus guaranteed optimal functionality that may need to be resolved. A novel screening method that links sequence functionality to fluorescence intensity was recently developed [53], which would demonstrate the effectiveness of functional aptamers *in vitro* evolution by obtaining modified DNA aptamers capable of disrupting the interactions in biological systems. We might also adopt this technique to check for our aptamers’ functionality involving their target association.

Following successes in our *in vitro* liposome binding assay experiments with selected aptamer candidates demonstrating their PS specific preferences, we are now also finalizing a plan on investigating the aptamers for their PS binding kinetics studies in molecular dynamics (MD) simulations [18,19,39] that might help measure both Coulomb and van der Waals energies borne out of charge-based interactions between drugs and target biomolecules [11,13]. MD simulations of the aptamer-PS complex will also let us understand how the long-range active charge-based interaction energetics dictate drug-target association/dissociation phenomena following a valid Statistical Mechanics formalism that we recently developed [39]. Our SCIA helps design aptamers by considering charge-based interactions of aptamers or ABBs and target biomolecules. Therefore, we predict to see a similar scenario to that reported in our previous studies [39].

The biological promise of targeting intracellular PS with extracellular aptamers may raise considerable questions. To clear this matter, we applied a technique to induce apoptosis into cancer cell lines to let PS molecules migrate to the cell surface in our previous studies [20]. We are also planning on measuring the PS binding potency of SCIA-designed aptamers, following the same techniques that we applied for EFBA-designed aptamers (see the details in ref [20]).

Our SCIA-based aptamer design technique enables the creation of sets of aptamer sequences for any target biomolecule of interest, thereby establishing a universal aptamer design platform. Other available bioinformatics frameworks, including AptaSuite [54,55], AptamerRunner [56], etc., that consider utilizing various advanced computational tools, machine learning algorithms, etc., may be used to cross-examine our SCIA-designed aptamers’ target binding potency and rank them regarding best performance predictions, besides our presented theoretical one here (see Fig. 6). In a biological target binding scenario, the drug molecules’ higher order structural moiety plays an important role. AlphaFold 3 [57] may be applied to understanding our designed aptamers’ 3D structures, considering them together with their target biomolecule(s) of interest; hence, we may finalize the high-performing optimal candidates before even going to rigorous *in vitro* or cytotoxicity biological assay experiments. Following the publication of these designed aptamer sequences (Table 5), we may later plan on these additional bioinformatics studies to enhance our understanding of the applicability of the aptamers in drug discovery, where PS is an obvious target in a disease (e.g., see Table 1).

Our discovered PS-binding aptamers (Table 5, large set or Fig. 7, specialized set) are candidates that may energetically show considerable PS binding potency. Therefore, these aptamer templates may be tried for discovering specific therapeutic or diagnostic drugs for diseases in which PS is the biomolecule of interest [[41], [42], [43], [44],^58^]. Besides, PS, being the candidate lipid that migrates across the apoptotic phase of any biological cell [59,60], it is vital to design agents that may detect PS externalization across the apoptotic phase as a result of chemotherapy drug-induced apoptosis [61] in cancer cells. Hence, our PS aptamers designed here and earlier [18,19] may emerge as drug agents capable of detecting the therapeutic induction of apoptosis in cancer cells, which will help us understand the efficacy of versatile chemotherapy drugs against corresponding cancer types [62,63]. Combining our PS aptamers as diagnostic drugs with available therapeutic drugs, we may help create a theranostic drug discovery template for chemotherapy applications.

## Conclusions

A generalized energy-based method, SCIA, has been developed, helping us design aptamers to target biomolecules in any biological system. PS-interacting aptamers have been discovered and validated for considerable aptamer-PS association in both *in silico* numerical computations and *in vitro* experiments. This is an extension of our previous studies with the same SCIA technique to design aptamers for two other lipids, PC [17] and ceramide (pending US patent), and a protein, Bcl-2 (pending US patent). SCIA thus emerged as a universal technology that utilizes fundamental physics principles, allowing for the design of aptamers for any biomolecule of interest. The PS aptamers that we have designed can be used to extend biomedical research to discovering drugs for diseases in which PS is directly involved or via its association with the cell membrane, creating physicochemical causes behind the rise of disease states.

## Ethical statement

Nothing to declare

## Declaration of competing of interest

None

## Data Availability

Data will be made available on request.

## Appendix Supplementary materials

Image, application 1
