# Supplementary material for: Energy-based method for designing aptamers to target phosphatidylserine
Source: MethodsX. 2026 Mar 19;16:103872. doi: 10.1016/j.mex.2026.103872 (PMC13068873; doi:10.1016/j.mex.2026.103872)
Supplement: Supplementary file 1 [file mmc1.docx]

**Energy-based method for designing aptamers to target phosphatidylserine**

Fatima Alharbi^1^, Hamed Alsulami^1^, Suliman AlOmar^2^, Md. Ashrafuzzaman^3,*^

^1^Biochemistry Department, College of Science, King Saud University, Riyadh, Saudi Arabia

^2^Doping Research Chair, Zoology Department, College of Science, King Saud University, Riyadh, Saudi Arabia

^3^Doping Research Chair, Deanship of Scientific Research, King Saud University, Riyadh, Saudi Arabia

*Corresponding author: [mzzaman@ksu.edu.sa](mailto:mzzaman@ksu.edu.sa); Tel.: +966 564174931

**Supplementary Materials**

*Derivation of binding energy, E*_SCI_

Ashrafuzzaman and Tuszynski [11,12] have described the potential for molecules to interact with each other as a multi-charge system, which was presumed in designing aptamers for targets in the biological system. Both target and aptamer are considered to consist of charges that interact among themselves as well as with other molecules, such as the case when the aptamer approaches the target. Thus, the interaction in the target aptamer complex is considered as a multi-charge system that follows SCI potential *V*_sc_ (k) as described in equation (1):


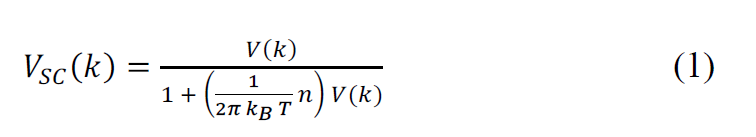


*k*_B_ is the Boltzmann constant (= 1.38 ×10−23 J/K), *T* is the absolute temperature for the thermodynamic condition of the system, and 𝑛 is the density of the participating particles, which in this case is the number or density of charges. *V(k)* is the direct Coulomb interaction (DCI) between two charges *q*_apt,_ which here is the value of a single charge of the nucleotide charge groups in an aptamer, *q*_tar_ is the value of a single charge in the charge distribution of the target, and follows the following relation (eq. 2):


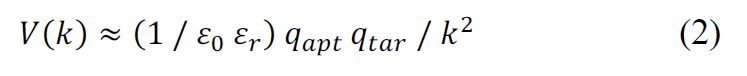


*ε*_0_ is the dielectric constant in a vacuum and *ε*_r_ (~80 in an aqueous water environment or ~2 in a hydrophobic environment like a membrane) is the relative dielectric constant of a medium, *k* is the wavenumber. Defining 𝑓(𝑇, 𝑛) as (eq. 3):


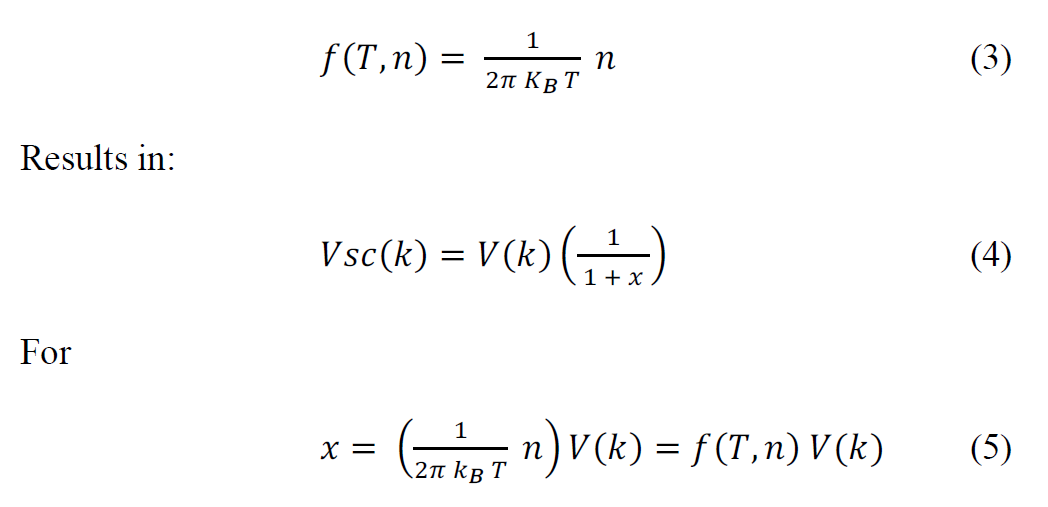


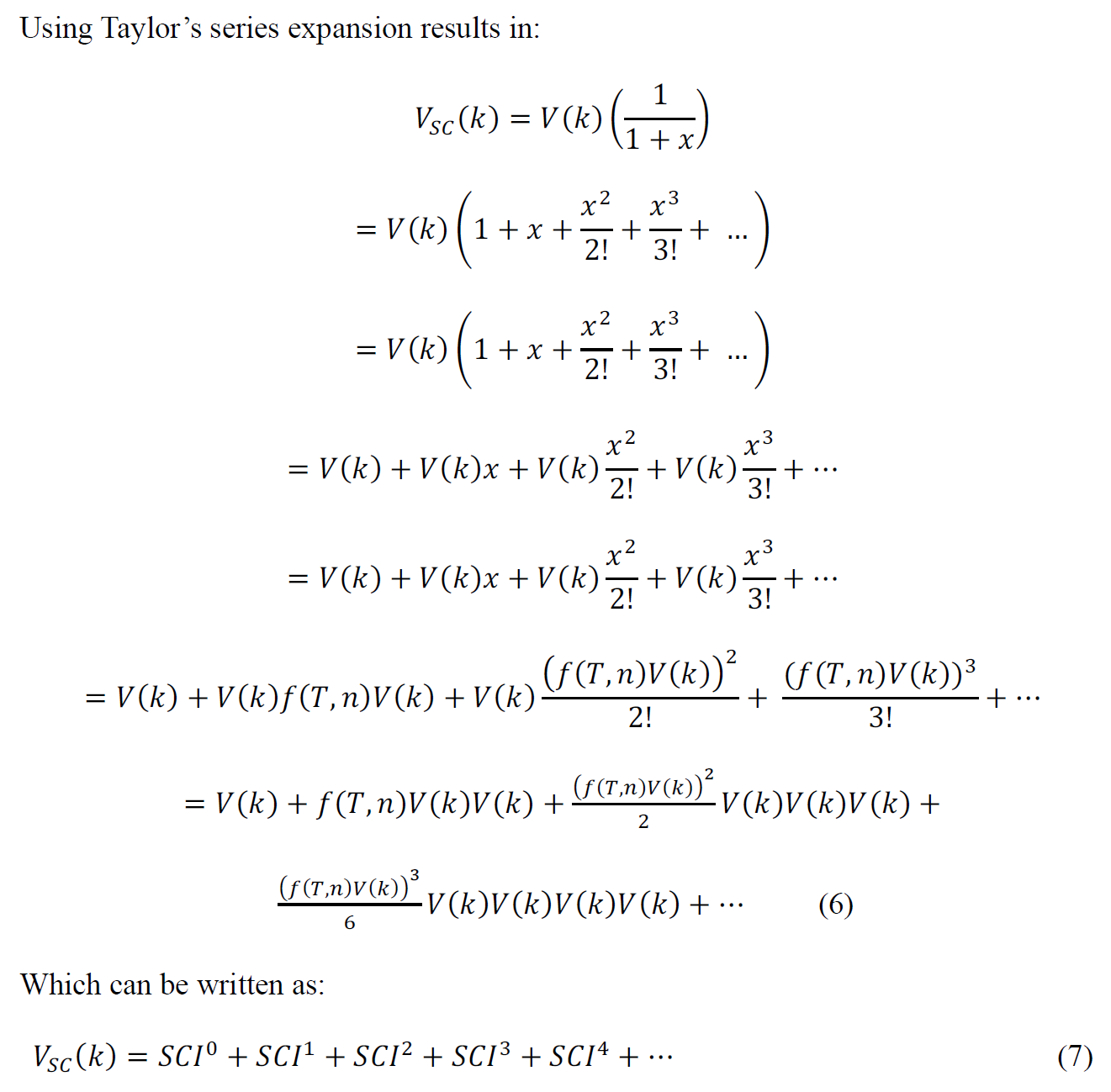


Here *SCI*^i^ (i = 0, 1, 2, 3, 4, . . ., etc.) represents the i^th^ order SCI with i = 0 representing the DCI interaction.

Following our strategic derivation in ref. [10,11,12], we see (eq. 8)

$V_{sc}(\vec{r})=\int d^{3}kExp\{i\vec{k}.\vec{r}\}V_{sc}(\vec{k})$ (8)

which is in Fourier space, $V_{Sc}(\vec{k})$ [11,12].

The Fourier transformation of equation (8) was performed using the Mathematica 9 program-based NCs (detailed methodologies can be found in refs. [11,12]) to detect the values of drug binding energy *E*_SCI_ and plotted against the reaction coordinate r (see Figs. 2-4). Following NCs, we get data for binding energy (e.g. see Fig. 3, generally modelled here in Fig. Supp. 1) showing transitions between higher energy (*G*_I_) and lower energy (*G*_II_) values and vice versa. The free energy of charge-charge association/dissociation is ΔG= ⏐*G*_I_ - *G*_II_⏐≈ ⏐*G*_I_⏐as in our data (e.g. Fig. 3), we see ⏐*G*_I_⏐is much higher than ⏐*G*_II_⏐.

**Figure ‎Supp. 1.** Energy (y-axis) versus reaction coordinate (x-axis) plot in any biomolecular association (11,12). I and II represent free and associated or bound states with higher and lower energies at I and II, respectively. Here Δ*G_PS,i_* ^I↔II^ = |*E_PS,i_*^I^ - *E_PS,i_*^II^|. In case of SCI energetics multiple Is represent for multiple SCI orders, namely, here I at lower layer represents for 1^st^ order SCI, upper layer one for 2^nd^ order SCI, etc.

In performing NCs, inter-particle/inter-charge separation (in SCIA), which is assumed here as the lattice constant, is a vital input parameter in algorithms [11,12]. Here, we calculate them for different cases. We present its detailed derivation strategy here.

*1D Modeling of Charge Distribution in PS-ABBs Complex*

The interactions between ceramide and ABBs are modeled in a 1D lattice to represent the interaction complexes and to calculate the lattice constant for each model.

1. *1D modeling of Charge Distribution in a Complex of a Single PS and a Single ABB*

Here the models represent a single PS with its charge distribution (3 charges) as dark brown-colored spheres interacting with a single ABB and its charge distribution, either three charges or four charges for A, T, and U or for C and G, respectively (Fig. Supp.2). For the purpose of demonstrating the length difference between purines and pyrimidines, PS-purine complex models are slightly longer than the other models (which represent the interactions complexes with pyrimidines).

**
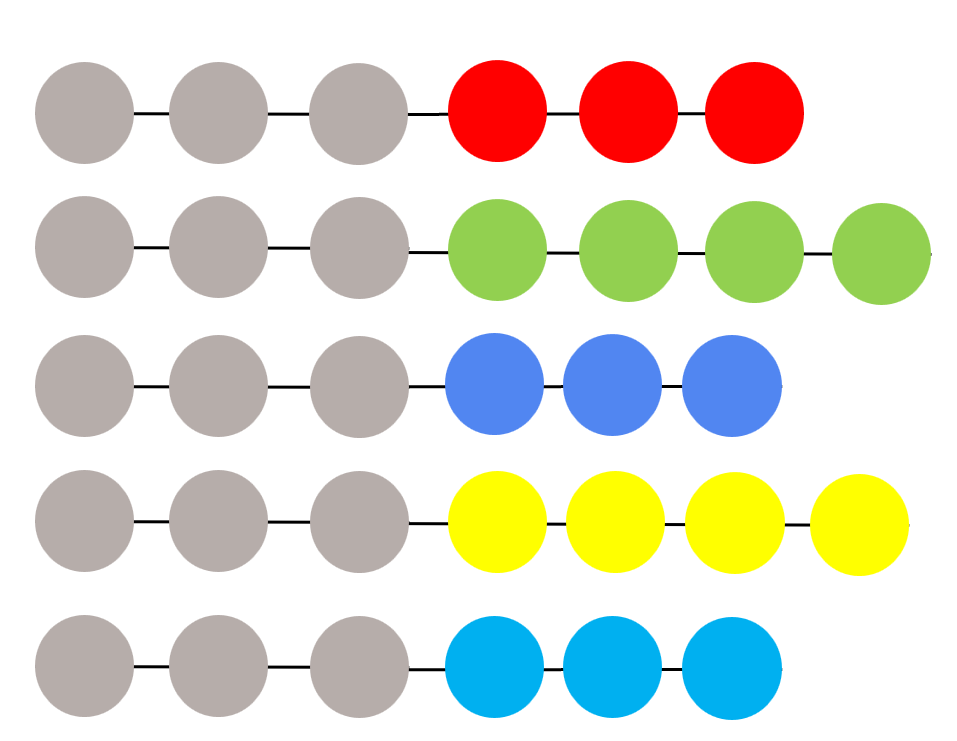
**

**Figure ‎Supp. 2.** The model structure of the charge distribution in a 1D lattice between PS (dark brown (leftmost), 3 charges) and ABBs (SCI interactions). Top to bottom: Charge distribution of the complexes of PS and A (red, 3 charges), G (green, 4 charges), T (blue, 3 charges), C (yellow, 4 charges), and U (light blue, 3 charges). Purine lattice constant (*a*_pu_) = 0.33 nm. Pyrimidine lattice constant (*a*_py_) = 0.3 nm. Their ratio (r_py/pu_) ≈ 0.9. Horizontal lengths among all represent variation in ABB chains due to variation in ABB dimensions.

The lattice constant for each model interaction was calculated using the following equation:

*a_0_* = $\frac{a_{PS} + a_{ABB}}{n_{0}}$

In our calculations, we assumed *a_py_ = r_py/pu_ a_pu_* with *r_py/pu_* ≤ 1. Let’s assume *r_py/pu_* = 0.9. *n*_0_ is the total number of charges in a PS-ABB complex. *a*_ABB_ is *a*_pu_ (≈ 0.33 nm [31]) or *a*_py_ (*r_py/pu_* *a_pu_* ≈ 0.3 nm). *a*_PS_≈ 1.0 nm (details in ref. [11]).

The calculated data are presented in Table Supp. 1.

**Table Supp. 1.** Calculated lattice constant for the zeroth order (SCI^0^) PS-ABB interactions [32,40,41].

| Complex | | *a*_0_ */ nm* |
| --- | --- | --- |
|  | PS-A | 0.222 |
|  | PS-G | 0.19 |
|  | PS-T | 0.217 |
|  | PS-C | 0.186 |
|  | PS-U | 0.217 |

1. *1D Modeling of Charge Distribution in a Complex of a Single PC and a Series of ABBs*

Here, PS (as a single charge molecule) interacts with a chain of ABBs, with each ABB having the same charge, depending on the ABB’s nucleotide type. This helps us calculate the lattice constant (*a*) of a PS and aptamer complex. When an aptamer contains $n_{ABB}$ nucleotides, the lattice constant *a* can be calculated as follows:

$$a= \frac{a_{PS}+n_{ABB}\times a_{ABB}}{n_{ABB}+1}$$

Where again we assume *a_py_ = r_py/pu_ a_pu_* with *r_py/pu_* ≤ 1. Let’s assume *r_py/pu_* = 0.9

Fig. Supp. 3 represents the 1D model (SCI interaction) of the complex of PS and aptamers of 5 ABBs ($n_{\mathrm{ABB}}$ = 5). The size difference in spheres represents the relative size difference between PS and any ABB. The lengths of the complexes are modelled to be different due to the fact that purines and pyrimidines appear with different geometrical dimensions.


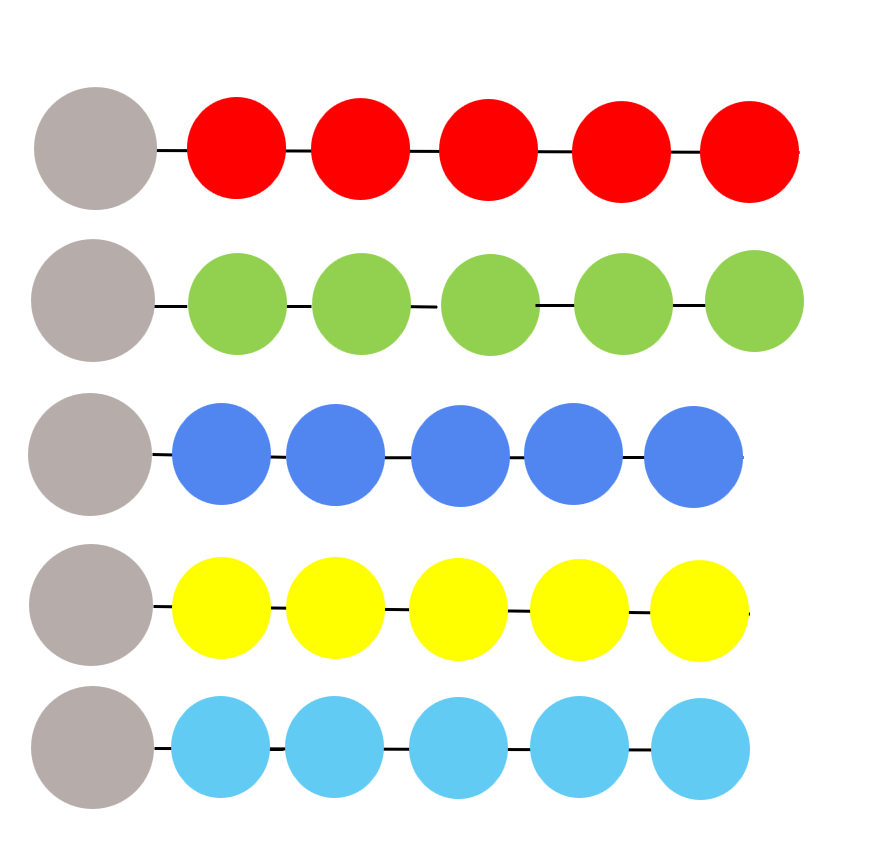


**Figure Supp. 3.** A 1D model structure of the interaction of PS (dark brown, leftmost spahere) with poly-purine or poly-pyrimidine aptamers (SCI interactions). The first two lines represent a PS aptamer complex with a chain of purines (A: red, G: green), the third-fifth lines represent a PS aptamer complex with chains of pyrimidines (T: blue, C: yellow, U: light blue).

We calculated the values of *a* (Table Supp. 2) for the cases of aptamers having 5 (Fig. Supp. 3), 6, or 10 ABBs. The choice of 5, 6 and 10 has been made to show that the value of the lattice constant varies with the length of the aptamer.

**Table Supp. 2.** Calculated lattice constant *a* for different cases of aptamer lengths, considering available information, see refs. [32,40,41] and our data for a similar case, where we addressed the design of PC binding aptamers [17].

| PS-Chain of ABB | *a/ nm*  (*n*_ABB_= 5) | *a/ nm*  (*n*_ABB_= 6) | *a/ nm*  (*n*_ABB_= 10) |
| --- | --- | --- | --- |
| A | 0.442 | 0.426 | 0.391 |
| G | 0.442 | 0.426 | 0.391 |
| T | 0.417 | 0.4 | 0.364 |
| C | 0.417 | 0.4 | 0.364 |
| U | 0.417 | 0.4 | 0.364 |

**Shell model to theoretically demonstrate target interaction of ABBs, leading to estimating possibility of aptamer design**

**Figure Supp. 4.** A shell model of drug target (brown sphere at the center of the shell) interactions (two-dimensional (2D) circular presentation of a spherical virtual shell). Here increasing interaction line thickness models for increasing SCI orders. Virtual circular lines (A) represent the possible uniform geometric locations where DBBs (B) may get distributed. For aptamers in this study, DBBs are ABBs of any nucleic acid type, e.g., DNA or RNA. For ref. see [10].

Figs. Supp. 4A and 4B depict a shell model of aptamer-target interactions (a 2D circle represents a spherical virtual shell). Increasing interaction line thickness represents increasing SCI order. Circular lines (Fig. Supp. 4A) represent the possible uniform geometric locations where ABBs (Fig. Supp. 4B) may be distributed.

Figs. Supp. 4A and 4B schematize the possible aptamers of different lengths and the contributions of SCI orders to the total SCI binding energy of the target-aptamer complex. In the particular example of ABBs selected from DNA nucleotides, for *m* DNA nucleotides in the sequence of an aptamer, there will 4*^m^* possible aptamers. Specific inter-molecular bond distances may be considered specific to each ABB-ABB and target ABB bond.

A matrix of ABBs for a specific target can be created from the combinatorial arrangements illustrated in Figs. Supp. 4A and 4B. Considering 4 possible ABBs for the case of DNA nucleotides, for example, there are 4*^m^* possible positions representing combinations, where *m*=1, 2, 3, …. , *m_max_*. The determination of *m_max_* is determined in view of the value of ABB-target SCI energy *E_i_* does not change between *i*=*m_max_*-2 and *i*=*m_max_*-1 (saturation of binding energy condition). This 4*^m^* represents the number of possible aptamers for a target with each having *m* number of ABBs. For DNA or RNA aptamers, 1<*m*≤*m_max_* because aptamers require at least 2 ABBs.

Exemplary numerical computation for calculation of *E_i_*

In general, for any single target structure if we consider *k* number of drug building blocks (DBBs) (here they are ABBs), we find that in each shell there are *k^m^* possible positions, accounting for the number drugs. The total number of drugs (*N_drug_*) (eq. 9),

$N_{drug}=\sum_{m=1}^{m_{max}} k^{m}$ (9)

But in case of aptamers, as explained earlier, we require a minimum 2 DBBs, so the total number of aptamers (*N_apt_*) (eq. 10),

$N_{apt}=\sum_{m=2}^{m_{max}} k^{m}$ (10)

For DNA or RNA aptamers, *k*=4 (equation 11), but for their (DNA and RNA) combinations, *k*=5.

In this combinatorial DBB arrangement, we find a lot of repeating sequences (considering forward and reverse sequences) that need to be excluded. E.g., AAG and GAA may be counted once. Thus, the actual number of candidate drugs or aptamers will be lower than *N_drug_* or *N_apt_*.
